# Association of *ACYP2* and *MPHOSPH6* genetic polymorphisms with the risk of hepatocellular carcinoma in chronic hepatitis B virus carriers

**DOI:** 10.18632/oncotarget.20846

**Published:** 2017-09-12

**Authors:** Yingai Zhang, Shunlan Wang, Xiaohong Wen, Shufang Zhang, Yijun Yang

**Affiliations:** ^1^ Central Laboratory, Haikou People’s Hospital, Central South University Xiangya School of Medicine Affiliated Haikou Hospital, Haikou 570208, Hainan, China; ^2^ Department of Hepatobiliary Surgery, Haikou People’s Hospital, Central South University Xiangya School of Medicine Affiliated Haikou Hospital, Haikou 570208, Hainan, China

**Keywords:** hepatocellular carcinoma (HCC), hepatitis B virus (HBV), *ACYP2*, *MPHOSPH6*, association analysis

## Abstract

Hepatocellular carcinoma (HCC) is the dominant histologic type of primary liver cancer, and hepatitis B virus (HBV) infection is one of the major causes of HCC in the chronic HBV. Our study was investigated the association between the polymorphisms of *ACYP2* and *MPHOSPH6* genes and the risk of HCC induced by HBV infection. A total of 490 subjects were divided into two groups: 248 HBV patients with HCC (Case group), and 242 HBV patients without HCC (Control group). Unconditional logistic regression analysis was used to evaluate the association. The genetic association analysis revealed variant of rs12621038 in *ACYP2* gene had a significant association with increasing the risk of HBV-induced HCC based on the genotype, dominant and additive model (*P*<0.05). Moreover, our results also showed that minor allele “C” of rs3751862 was prevalent in cases than controls (*P*<0.05), and rs3751862 significantly increased the risk of HCC in chronic HBV carriers under genotype and dominant model (*P*<0.05). In addition, the haplotype “T-G-G” in *MPHOSPH6* showed a harmful factor for the HBV-induced HCC (*P*<0.05). The results suggested that *ACYP2* and *MPHOSPH6* as the plausible candidate genes may predict the risk of HCC after chronic HBV infection in Chinese Han population, and further investigations in studies with a larger sample size and other races are needed to validate our findings. These data provide a theoretical foundation for future studies of this correlation between the polymorphisms of *ACYP2* and *MPHOSPH6* genes and the HCC in chronic HBV carriers.

## INTRODUCTION

Liver and intrahepatic bile duct cancer is the fifth most common diagnosed cancer and ranks eighth among causes of cancer mortality worldwide, accounting for approximately 41,000 new cancer cases and 29,000 deaths in the United States in 2017 [1]. In China, liver cancer is also the most common etiology of the cancer death [2]. About 70%-90% of the primary liver cancers is hepatocellular carcinoma (HCC), which is a complex and multi-factorial related disease [3, 4], and many evidences have revealed that chronic infection with hepatitis B virus (HBV) and hepatitis C virus (HCV) contribute to the major risk factor for HCC globally [5, 6]. Furthermore, areas with a high prevalence of HBV infection such as southeastern Asia, African, HBV is the most common etiologic agent of HCC [7].

Epidemiological studies have demonstrated a 5 to 10-fold increase in the relative risk of HCC among HBV carriers compared to noncarriers [3]. HBV, a family of enveloped viruses with an incomplete double stranded DNA genome of 3.2 kb, belongs to hepadnaviridae. The genome of HBV is composed of four partially overlapping open reading frames, which encode the hepatitis B antigens, viral polymerase and multifunctional nonstructural protein. In the present study, many studies suggested that HBV genotypes, viral load, and viral mutations were closely associated with the occurrence of HCC [8, 9]. Additionally, host genetic variations played a crucial role in the occurrence and development of HBV related HCC [10, 11]. On the other hand, a genome wide association study (GWAS), demonstrated that 1p36.22 as a new susceptibility loci cluster for HCC in chronic HBV carriers were identified, and these data suggested that *KIF1B*, *UBE4B* or *PGD* might be a plausible causative genes for this malignancy in the Chinese population [12]. Consequently, seeking for some chronic HBV-related HCC susceptible sites has attracted many scholars’ attentions.

Presently, relative telomere length has emerged as a promising risk precursor of many cancers containing HCC [13-15], and GWAS studies have identified that *ACYP2* is an important telomere length related gene [16]. *ACYP2* gene, located on chromosome 2p16.2, encodes small cytosolic acylphosphatase enzyme, and publication have explored the association between *ACYP2* polymorphisms liver cancer risk [17]. In addition, an association study has explored the polymorphisms of *MPHOSPH6* on the risk of colorectal cancer [18]. *MPHOSPH6* gene, located on chromosome 16q23.3, encodes M-phase phosphoprotein 6 might be involved in regulating cell cycle [19]. However, the association of *ACYP2* and *MPHOSPH6* genetic polymorphisms with risk of HCC in chronic HBV carriers was not clear in Chinese Han population. Therefore, in this study, we aimed to explore the effect of *ACYP2* and *MPHOSPH6* variations in the progression of chronic HBV induced HCC in Chinese Han population, and twelve SNPs have been selected from the two candidate genes.

## RESULTS

### Characteristic of the study participants

Demographic and epidemiological characteristics of the participants were summarized in Table 1. There was no significant difference in gender between the two groups (*P*=0.944), however, there was a significant difference between the case and control in mean age (54.47±12.05 *vs* 50.04±12.05, *P*<0.001). In this study, we also investigated the effects of cigarette smoking and alcohol consumption, but a considerable number of patients was not clear. Consequently, unconditional logistic regression analysis was performed adjusting for gender, age, cigarette smoking and alcohol consumption.

**Table 1:** Distributions of select variables in hepatocellular carcinoma patients and hepatitis B patients

Variables	Case	Control	*P* value
Gender			0.944^a^
Male (%)	192 (77.4%)	188 (77.7%)	
Female (%)	56 (22.6%)	54 (22.3%)	
Age			
Mean age ± SD (years)	54.47±12.05	50.04±12.05	<0.001^b^
Cigarette smoking			
Non-smoker (%)	156 (62.9%)	116 (47.9%)	<0.001^a^
Smoker (%)	43 (17.3%)	126 (52.1%)	
Missing (%)	49 (19.8%)		
Alcohol consumption			
Non-drinker (%)	160 (64.5%)	152 (62.8%)	<0.001^a^
Drinker (%)	26 (10.5%)	90 (37.2%)	
Missing (%)	62 (25%)		

SD: standard deviation. *P*^**a**^-value was calculated by Pearson’s χ^2^ test, and *P*^**b**^–value was calculated by Welch’s t test.

### Genotype model analysis

Table 2 summarized the basic information and miner allelic frequency of tested SNPs among individuals between the cases and controls. All 12 SNPs were in Hardy-Weinberg equilibrium (HWE) in control subjects (*P*> 0.05). We compared the difference in frequency distributions of alleles by Pearson’s χ^2^ test and found that minor allele “C” of rs3751862 in *MPHOSPH6* was more prevalent in case than control (OR=1.99, 95%CI=1.04-3.83, *P*=0.036). However, no significant association was found between other SNPs and the risk of HCC in chronic HBV carriers.

**Table 2:** Genotype and allele frequencies of candidate SNP loci in cases and controls

SNP rs#	Position	Band	Gene(s)	Role	Alleles A/B	HWE-*P*^a^	Minor Allele Frequency		OR(95%CI)	*P*^b^
							Case	Control		
rs6713088	54345469	2p16.2	ACYP2	Intron	G/C	0.6227	0.442	0.414	1.12 (0.87-1.44)	0.376
rs12621038	54391113	2p16.2	ACYP2	Intron	T/C	0.4149	0.482	0.423	1.27 (0.98-1.63)	0.067
rs1682111	54427979	2p16.2	ACYP2	Intron	A/T	0.6043	0.297	0.348	0.79 (0.6-1.04)	0.088
rs843752	54446587	2p16.2	ACYP2	Intron	G/T	0.8774	0.267	0.267	1 (0.75-1.33)	0.992
rs10439478	54459450	2p16.2	ACYP2	Intron	C/A	0.2374	0.433	0.383	1.23 (0.95-1.59)	0.111
rs17045754	54496757	2p16.2	ACYP2	Intron	C/G	0.5465	0.202	0.159	1.33 (0.96-1.85)	0.084
rs843720	54510660	2p16.2	ACYP2	Intron	G/T	0.4511	0.316	0.355	0.84 (0.64-1.1)	0.199
rs1056675	82181934	16q23.3	MPHOSPH6	3’ UTR	C/T	0.8104	0.42	0.424	0.99 (0.76-1.27)	0.909
rs1056654	82182011	16q23.3	MPHOSPH6	3’ UTR	A/G	0.5845	0.278	0.299	0.9 (0.69-1.19)	0.479
rs3751862	82182229	16q23.3	MPHOSPH6	3’ UTR	C/A	0.4074	0.056	0.029	1.99 (1.04-3.83)	0.036*
rs11859599	82182832	16q23.3	MPHOSPH6	Intron (boundary)	C/G	0.0899	0.241	0.244	0.98 (0.73-1.32)	0.914
rs2967361	82203503	16q23.3	MPHOSPH6	Intron	T/G	0.07924	0.256	0.211	1.29 (0.96-1.74)	0.094

SNPs: Single nucleotide polymorphisms; A: Minor alleles, B: Major alleles; HWE: Hardy-Weinberg equilibrium; OR: Odds ratio. CI: Confidence interval.

*P*^**a**^-values were calculated using exact test; *P*^**b**^-values were calculated using Chi-square test.

*: *P* value < 0.05 indicates statistical significance.

We hypothesized that harboring the minor allele of each SNP was regarded as a risk factor compared with the wild type allele. Comparison of the SNP genotypes and the risk of HCC were assessed by logistic regression test adjusted for gender, age, cigarette smoking and alcohol consumption in Table 3 and Table 4. Four genetic models (genotype, dominant, recessive and additive model) were applied to analyze the correlation. In Table 3, we found that the minor allele “T” of rs12621038 in *ACYP2* was significantly associated increasing the risk of HCC, based on the genotype model “C/T” in chronic HBV carriers increasing a 2.14-flod risk for inducing HCC compared to the “C/C” genotype (95%CI=1.27-3.62, *P*=0.004). And compared with genotype “A/A”, the genotype “A/C” of rs3751862 in *MPHOSPH6* also significantly associated increasing 2.57-flod the risk of HBV-related HCC (95%CI=1.13-5.83, *P*=0.024). Additionally, from the result of Table 4, rs12621038 was found increasing the risk of HCC in chronic HBV carriers under dominant model (OR=2.05, 95%CI=1.25-3.56, *P*=0.004), and additive model (OR=1.41, 95%CI=1.03-1.93, *P*=0.032). As for rs3751862, under dominant model, was found correlating with a 2.39-fold higher risk of HCC at the 5% level (95%CI=1.07-5.31, *P*=0.033). However, SNPs except rs12621038 and rs3751862 were not been found any significant result in the genotypic model.

**Table 3:** Association between polymorphism of candidates SNPs and the HBV-related HCC risk under genotype model

SNP	Allele A/B	Genotype	Genotype Frequency		Logistic regression		
			Case	Control	OR	95%CI	*P*
rs6713088	G/C	C/C	29%	34.6%	1	-	-
		C/G	53.6%	48.1%	1.6	0.98-2.63	0.062
		G/G	17.3%	17.3%	0.92	0.47-1.79	0.798
rs12621038	T/C	C/C	25.6%	36.5%	1	-	-
		C/T	52.4%	42.3%	2.14	1.27-3.62	0.004*
		T/T	22%	21.2%	1.85	0.98-3.5	0.058
rs1682111	A/T	T/T	50.8%	44.4%	1	-	-
		T/A	39%	41.6%	0.85	0.53-1.36	0.498
		A/A	10.2%	14%	0.54	0.27-1.09	0.086
rs843752	G/T	T/T	53%	54.3%	1	-	-
		T/G	40.5%	37.9%	1.27	0.8-2.02	0.308
		G/G	6.5%	7.8%	0.59	0.24-1.5	0.268
rs10439478	C/A	A/A	32%	37.7%	1	-	-
		A/C	49.4%	48%	1.21	0.75-1.98	0.436
		C/A	18.6%	14.3%	1.85	0.95-3.62	0.071
rs17045754	C/G	G/G	63.7%	71.1%	1	-	-
		G/C	32.3%	26%	1.18	0.73-1.92	0.501
		C/C	4%	2.9%	1.43	0.4-5.16	0.584
rs843720	G/T	T/T	45.7%	43.4%	1	-	-
		T/G	45.3%	42.2%	0.94	0.59-1.51	0.813
		G/G	8.9%	14.3%	0.8	0.38-1.69	0.563
rs1056675	C/T	T/T	33.9%	32.1%	1	-	-
		T/C	48.2%	51.1%	0.99	0.59-1.65	0.97
		C/C	18%	16.9%	1.24	0.64-2.4	0.533
rs1056654	A/G	G/G	52.8%	48.1%	1	-	-
		G/A	38.7%	44%	0.63	0.4-1.01	0.055
		A/A	8.5%	7.9%	0.92	0.39-2.15	0.842
rs3751862	C/A	A/A	88.7%	94.6%	1	-	-
		A/C	11.3%	5%	2.57	1.13-5.83	0.024*
		C/C	0	0.4%	-	-	-
rs11859599	C/G	G/G	59.9%	57%	1	-	-
		G/C	32%	37.3%	0.83	0.52-1.33	0.436
		C/C	8.1%	5.7%	1.26	0.48-3.29	0.643
rs2967361	T/G	G/G	55.2%	62.4%	1	-	-
		G/T	38.3%	33.1%	1.26	0.79-2	0.336
		T/T	6.5%	4.5%	1.06	0.36-3.12	0.923

SNPs: Single nucleotide polymorphisms; A: Minor alleles, B: Major alleles; OR: Odds ratio. CI: Confidence interval.

*P***–**value was calculated by unconditional logistic regression adjusted for gender, age, cigarette, smoking and alcohol consumption.

*P value < 0.05 indicates statistical significance.

**Table 4:** Association of SNPs polymorphisms with the HBV-related HCC risk under dominant, recessive and additive model

SNP	Dominant model			Recessive model			Additive model		
	OR	95%CI	*P*	OR	95%CI	*P*	OR	95%CI	*P*
rs6713088	1.39	0.87-2.23	0.165	0.69	0.38-1.27	0.236	1.05	0.77-1.45	0.75
rs12621038	2.05	1.25-3.36	0.004*	1.16	0.67-1.99	0.6	1.41	1.03-1.93	0.032*
rs1682111	0.76	0.49-1.19	0.231	0.58	0.3-1.14	0.113	0.77	0.56-1.06	0.103
rs843752	1.13	0.73-1.76	0.578	0.54	0.22-1.33	0.18	0.98	0.69-1.39	0.91
rs10439478	1.35	0.85-2.13	0.208	1.66	0.9-3.03	0.102	1.33	0.96-1.84	0.082
rs17045754	1.2	0.75-1.92	0.44	1.36	0.38-4.87	0.636	1.19	0.79-1.78	0.411
rs843720	0.91	0.58-1.43	0.692	0.82	0.4-1.68	0.595	0.91	0.65-1.27	0.586
rs1056675	1.05	0.64-1.71	0.848	1.24	0.7-2.22	0.462	1.1	0.79-1.52	0.587
rs1056654	0.67	0.43-1.05	0.08	1.12	0.49-2.56	0.786	0.8	0.56-1.13	0.204
rs3751862	2.39	1.07-5.31	0.033*	0	-	-	2.1	0.99-4.45	0.054
rs11859599	0.88	0.56-1.38	0.577	1.34	0.52-3.47	0.544	0.96	0.67-1.38	0.824
rs2967361	1.23	0.79-1.93	0.361	0.97	0.33-2.83	0.956	1.16	0.79-1.69	0.451

ORs: odds ratios; CI: Confidence Interval.

*P***-**value was calculated by unconditional logistic regression adjusted for gender, age, smoking and drinking.

*P value < 0.05 indicates statistical significance.

### Haplotype analysis

**Figure 1:**
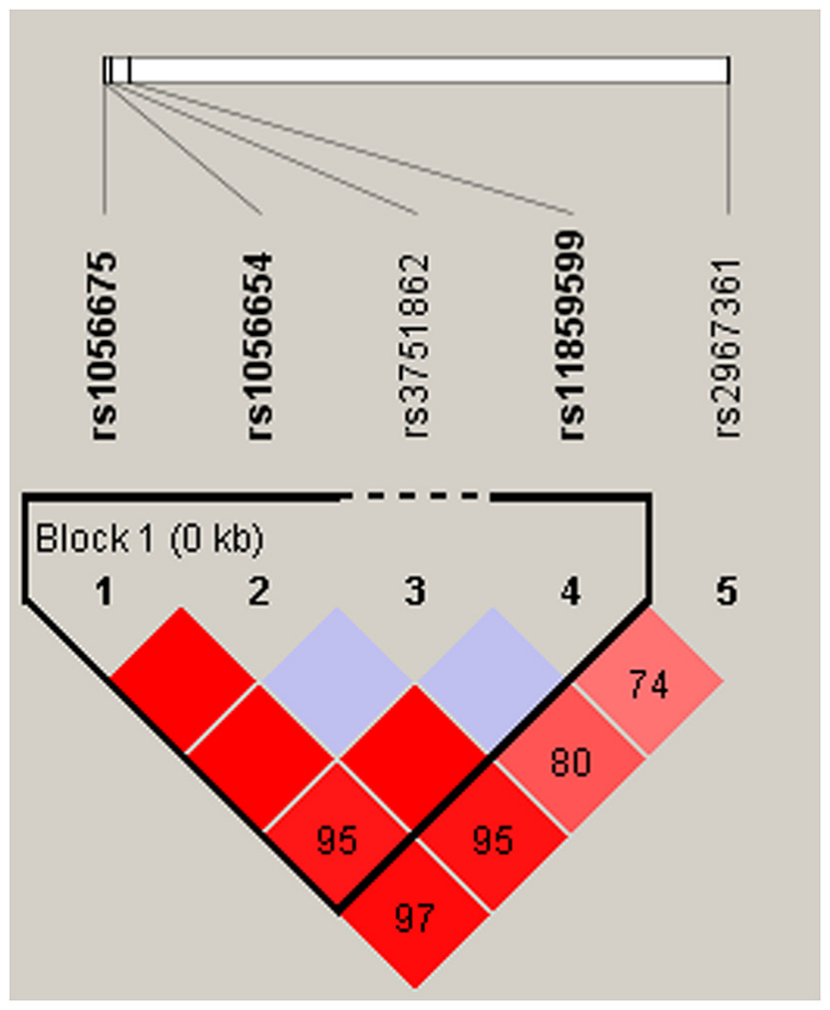
Haplotype block map for part of the SNPs in *MPHOSPH6* gene Linkage disequilibrium plots containing five SNPs from 16q23.3. Standard color frame is used to show LD pattern. Three SNPs rs1056675, rs1056654 and rs11859599 from the map are indicated a higher level of LD, and D’ value is 0.95.

Finally, we used the allele frequency data from all the subjects to do an LD analysis. Only one block containing three SNPs (rs1056675, rs1056654 and rs11859599) in *MPHOSPH6* was detected with D’=0.95 (Figure 1). The associations between the *MPHOSPH6* haplotypes and the risk of HCC in chronic HBV carriers were listed in Table 5. We found that the “T-G-G” haplotype was significantly associated with the risk of HCC by Pearson’ χ^2^ test (*P*=0.019). Furthermore, it was remained significantly augmenting the risk after unconditional logistic regression analysis adjusted for gender, age, cigarette smoking and alcohol consumption (OR=2.21, 95%CI=1.03-4.77, *P*=0.043). No association could be found between any other haplotypes from the block and the risk of HBV-related HCC.

**Table 5:** The haplotypes of three SNPs (rs1056675, rs1056654 and rs11859599) in *MPHOSPH6* and the HBV-related HCC risk

Haplotype	Freq (control)	Freq (case)	Chi-square	*P*^a^	OR	95%CI	*P*^b^
T-G-C	0.245	0.241	0.021	0.885	0.95	0.66-1.37	0.784
T-A-G	0.298	0.279	0.431	0.512	0.8	0.57-1.15	0.228
C-G-G	0.43	0.423	0.049	0.826	1.08	0.78-1.49	0.646
T-G-G	0.027	0.057	5.475	0.019*	2.21	1.03-4.77	0.043*

*P*^**a**^**-**value was calculated from two-sided Chi-squared test. *P*^*b*^-value was calculated by unconditional logistic regression adjusted for gender, age, cigarette smoking and alcohol consumption.

*P value <0.05 indicates statistical significance.

## DISCUSSION AND CONCLUSION

In our study, we investigated the associations between 12 SNPs of *ACYP2* and *MPHOSPH6* and the risk of HCC in chronic HBV carriers in Chinese Han population. We determined the minor allele “C” of rs3751862 in *MPHOSPH6* was associated with an increased risk of HBV-related HCC under allelic, genotype and dominant model. Moreover, we determined that the minor allele (T) of rs12621038 in *ACYP2* was associated with an increased risk of HBV-related HCC according to the genotypic model analysis, containing genotype, dominant, and additive model. In addition, we demonstrated that “A-G-G” haplotype in *MPHOSPH6* was associated with an increased risk of HCC surpass 2-fold in chronic HBV carriers.

So far as we know rs3751862 is located in the 3’ UTR of *MPHOSPH6*, however, little is known about the association between rs3751862 and the risk of diseases. *MPHOSPH6*, which localizes in the nucleus during interphase, was firstly identified by the characteristic of its phosphorylation during mitosis, and then found to be a M phase-related gene in mitotic cell cycle [20]. Previous studies have demonstrated that *MPHOSPH6* played a role in on the maturation of 5.8S rRNA as a nucleolus specific exosome factor [21]. Furthermore, the expression of *MPHOSPH6* had a significantly difference between oral squamous cell carcinoma samples and their corresponding surgical margins [22]. In our study, we found that rs3751862 was a risk variant for HCC in chronic HBV patients, and the haplotype “T-G-G” (rs1056675, rs1056654 and rs11859599 block) also be a harmful factor for the risk of chronic HBV related HCC. It might a role in a role in hypothesized that the mutation of this locus might alter the effect of microRNA on the regulation of the *MPHOSPH6*, resulting in abnormal cell division, and the mechanistic details should be identified in future studies.

The SNP rs12621038 locates in the intron of *ACYP2.* Previous studies have identified that genetic variants in *ACYP2* could be a risk factor for the etiology of many diseases. Recently, some, studies found SNPs in *ACYP2* were associated with many cancers, for example rs12621038, rs1682111 and rs17045754 could significantly decreased the risk of breast cancer [23], and rs843720 was associated with increased the lung cancer risk [24]. Furthermore, polymorphism of rs17045754 was related to increased risk of developing ischemic stroke [25]. Additionally, an association study revealed that polymorphism of rs6713088 was associated with an increased risk of liver cancer [17]. However, there is lack of association between polymorphisms of *ACYP2* and the susceptibility of HCC in chronic HBV carriers. We found rs12621038 might be a harmful factor significantly increased the development of HCC after HBV infection. Acylposphatase, encoded by *ACYP2*, can hydrolyze the phosphoenzyme intermediate of different membrane pumps, particularly the Ca^2+^/Mg^2+^-ATPase from sarcoplasmic reticulum of skeletal muscle [26]. Although the association mechanism is unclear, and future prospective multicenter study should be validated.

We acknowledged that there were several limitations in this study. On one hand, we cannot obtain the accurate years that the patients had been infected with HBV, furthermore we also could not precisely determine the time when chronic HBV carriers turned out to be the HCC patients. On the other hand, we didn’t have a relatively larger sample size, and alcohol consumption and cigarette smoking status of many patients in case group was absent. Thus, the associations between these confounding factors and the risk of HCC in chronic HBV carriers were not analyzed. We will evaluate these associations in the future.

In conclusion, our case control study explored the variants of *ACYP2* and *MPHOSPH6* with HCC susceptibility in chronic HBV carriers, and our results suggested that *MPHOSPH6* and *ACYP2* as a plausible candidate genes predicted the risk of HCC after chronic HBV infection in Chinese Han population. The findings need further confirmed in a larger cohort of HBV-induced HCC patients of other races. In addition, there is an urgent need to reveal the mechanism underlying the associations between *MPHOSPH6* and *ACYP2* and the risk of HCC induced by chronic HBV infection.

## MATERIALS AND METHODS

### Subject recruitment and ethics committee statement

This study was performed using two independent case-control populations. A total of 490 participants from the Haikou People’s Hospital were consecutively recruited between July 2014 and March 2016, among them 248 individuals were chronic HBV patients with HCC as the case group, and the remaining 242 persons were chronic HBV patients without HCC as the control group. All cases and controls selected for this study were confirmed by being positive for HBV surface antigen, HBV core antibody, hepatitis B e-antigen or hepatitis B e-antibody at least 6 months. Additional, that individuals contained in study were negative for hepatitis C surface antigen, human immunodeficiency virus. The HCC cases were diagnosed based on pathological identification combined with at least one positive liver image on computed tomography, magnetic resonance imaging, or ultrasonography, occasionally combined with increased serum AFP levels (>400ng/mL) [27, 28]. The controls were examined without any cancer.

Informed written consent was given by all participants, and some information of participates also collected, including age, gender, smoking and drinking status. This study was approved by the ethics committee of the Central South University Xiangya School of Medicine and the ethics committee of Haikou People’s Hospital. All clinical assessment and specimen collections were conducted according to Declaration of Helsinki principles.

### SNP selection and genotyping

Peripheral whole blood was collected from each subject and placed into anticoagulative tubes at -80°C. According to the Gold Mag-Mini extraction method (GodMag Co. Ltd, Xi’an, China) [29], genomic DNA extracted from whole blood. DNA concentration and purity were measured by spectrometry (NanoDrop 2000, Thermo Fisher Scientific, Waltham, Massachusetts, USA), and genomic DNA concentration and purity of all samples met the experimental requirements. Single base extensive primers were designed by Sequenom MassARRAY Assay Design 3.0 Software (San Diego, California, USA). Genotyping was performed using a Sequenom MassARRAY RS1000 (Sequenom, Inc., San Diego, CA, USA) according to the manufacturer’s instructions [30]. Sequenom Typer 4.0 software was used to performed genotyping data analysis and management [31].

In this study, seven SNPs in the *ACYP2* gene were selected from the publication that associated polymorphisms with liver cancer in Chinese Han population [17], including s6713088, rs12621038, rs1682111, rs843752, rs10439478, rs17045754 and rs843720. And five SNPs rs1056675, rs1056654, rs371862, rs11859599 and rs2967361 in *MPHOSPH6* were also selected according to the previous association study [18]. The SNPs were selected based on the minor allele frequencies of more than 5% in the HapMap Chinese Han population.

### Statistical analysis

Statistical analyses were performed using SPSS version 17.0 (SPSS, Chicago, IL, USA). All statistical tests were two sides, and *P*<0.05 indicated a significant difference. Pearson’s χ^2^ test was used to examine the statistical differences of categorical variables, containing gender, smoking status and drinking status; and Welch’s test was used to detect the statistical difference of continuous variable, such as age. The control group needed to detect whether the Hardy-Weinberg Equilibrium (HWE) met, and exact test was used to determine the SNPs departed from the HWE. Afterwards, the association between minor allele of each SNPs and the risk of HCC in chronic HBV carriers were evaluated by Pearson’s χ^2^ test, and the genotype model also constructed to assess the association. Finally, SHEsis software platform [32] and Haploview software package (version 4.2) (Broad Institute, Cambridge, MA, USA) [33] were used to construct the linkage disequilibrium (LD) block, and analyse the association between haplotypes and the risk of HCC. Unconditional logistic regression analysis adjusted for gender, age, smoking and drinking was used to calculate the Odds ratios (OR) and 95% confidence intervals (95%CI) for each variant.

## Abbreviations

HCC: Hepatocellular carcinoma
HBV: Hepatitis B virus
SNP: Single nucleotide polymorphism
OR: Odds ratio
CI: Confidence interval
LD: Linkage disequilibrium
HWE: Hardy-Weinberg Equilibrium
